# Use of Ebola Vaccine: Recommendations of the Advisory Committee on Immunization Practices, United States, 2020

**DOI:** 10.15585/mmwr.rr7001a1

**Published:** 2021-01-08

**Authors:** Mary J. Choi, Caitlin M. Cossaboom, Amy N. Whitesell, Jonathan W. Dyal, Allison Joyce, Rebecca L. Morgan, Doug Campos-Outcalt, Marissa Person, Elizabeth Ervin, Yon C. Yu, Pierre E. Rollin, Brian H. Harcourt, Robert L. Atmar, Beth P. Bell, Rita Helfand, Inger K. Damon, Sharon E. Frey

**Affiliations:** ^1^National Center for Emerging and Zoonotic Infectious Diseases, CDC; ^2^Department of Health Research Methods, Evidence, and Impact, McMaster University Hamilton, Ontario; ^3^University of Arizona College of Public Health, Tucson, Arizona; ^4^Baylor College of Medicine, Houston, Texas; ^5^University of Washington, Seattle, Washington; ^6^Saint Louis University School of Medicine, St. Louis, Missouri

## Abstract

This report summarizes the recommendations of the Advisory Committee on Immunization Practices (ACIP) for use of the rVSVΔG-ZEBOV-GP Ebola vaccine (Ervebo) in the United States. The vaccine contains rice-derived recombinant human serum albumin and live attenuated recombinant vesicular stomatitis virus (VSV) in which the gene encoding the glycoprotein of VSV was replaced with the gene encoding the glycoprotein of Ebola virus species *Zaire ebolavirus*. Persons with a history of severe allergic reaction (e.g., anaphylaxis) to rice protein should not receive Ervebo. This is the first and only vaccine currently licensed by the Food and Drug Administration for the prevention of Ebola virus disease (EVD). These guidelines will be updated based on availability of new data or as new vaccines are licensed to protect against EVD.

ACIP recommends preexposure vaccination with Ervebo for adults aged ≥18 years in the U.S. population who are at highest risk for potential occupational exposure to Ebola virus species *Zaire ebolavirus* because they are responding to an outbreak of EVD, work as health care personnel at federally designated Ebola treatment centers in the United States, or work as laboratorians or other staff at biosafety level 4 facilities in the United States. Recommendations for use of Ervebo in additional populations at risk for exposure and other settings will be considered and discussed by ACIP in the future.

## Introduction

On December 19, 2019, the rVSVΔG-ZEBOV-GP Ebola vaccine (Ervebo), a replication-competent, live attenuated vaccine was approved by the Food and Drug Administration (FDA) for the prevention of Ebola virus disease (EVD) caused by Ebola virus species *Zaire ebolavirus* (EBOV) in adults aged ≥18 years. The vaccine contains rice-derived recombinant human serum albumin and contains live attenuated recombinant vesicular stomatitis virus. The recombinant virus was created by replacing the gene encoding the glycoprotein of the vesicular stomatitis virus strain *Indiana* with the gene encoding the glycoprotein of the EBOV-Kikwit 1995 strain. At the time of Ervebo licensure, the Advisory Committee for Immunization Practices (ACIP) had no recommendations for the use of vaccines to prevent EVD. On February 26, 2020, ACIP recommended preexposure vaccination with Ervebo for adults aged ≥18 years in the United States who are at highest risk for potential occupational exposure to EBOV because they are responding to an outbreak of EVD, work as health care personnel* at federally designated Ebola treatment centers in the United States, or work as laboratorians or other staff at biosafety level 4 facilities in the United States (Box).

BOX **Institutions with federally designated Ebola treatment centers and biosafety level 4 facilities in the United States as of December 2020**

**Institutions with federally designated Ebola treatment centers**

- Emory University, Atlanta, Georgia
- Nebraska Medical Center, Omaha, Nebraska
- NYC Health + Hospitals/Bellevue, New York, New York
- Denver Health Medical Center, Denver, Colorado
- Johns Hopkins Hospital, Baltimore, Maryland
- Cedars-Sinai Medical Center, Los Angeles, California
- University of Minnesota Medical Center, Minneapolis, Minnesota
- Providence Sacred Heart Medical Center and Children’s Hospital, Spokane, Washington
- University of Texas Medical Branch at Galveston, Galveston, Texas
- National Institutes of Health, Bethesda, Maryland
- Massachusetts General Hospital, Boston, Massachusetts

**Biosafety level 4 facilities**

- CDC, Atlanta, Georgia
- Galveston National Laboratory, Galveston, Texas
- Georgia State University, Atlanta, Georgia
- Shope Laboratory, Galveston, Texas
- Integrated Research Facility, Frederick, Maryland
- Texas Biomedical Research Institute, San Antonio, Texas
- U.S. Army Medical Research Institute of Infectious Diseases, Frederick, Maryland
- Rocky Mountain Laboratories, Hamilton, Montana
- National Emerging Infectious Disease Laboratories, Boston, Massachusetts
- National Biodefense Analysis and Countermeasures Center, Frederick, Maryland

## Background

With case-fatality rates of 70%–90% when untreated, EBOV is the most lethal of the four viruses within the family *Filoviridae* that cause EVD in humans (*1*). EBOV is responsible for the majority of reported EVD outbreaks in humans (19 of 32^†^; 59%), including the two largest EVD outbreaks in history (*2*). 

EBOV is an enveloped, nonsegmented, negative-stranded RNA virus. The genome of EBOV encodes seven viral structural proteins: one glycoprotein, one nucleoprotein, one RNA-dependent RNA polymerase, and four virion proteins. The glycoprotein is the only transmembrane surface protein of the virus and is critical for attachment to host cells and cell membrane fusion (*3*).

Information about the pathology and pathogenesis of EBOV infections in humans is limited. However, studies in experimentally infected nonhuman primates have demonstrated that EBOV has a broad cell tropism. Monocytes, macrophages, and dendritic cells appear to be early and preferred targets and have an important role in viral dissemination (*1*,*4*). Virus-induced release of cytokines and chemokines results in dysregulation of the immune system and, in certain cases, disseminated intravascular coagulation and multisystem organ failure (*1*).

The natural animal reservoir for EBOV is not known; however, several studies have implicated bats. EBOV has been detected by real-time reverse transcriptase–polymerase chain reaction (rRT-PCR) and serology in three species of fruit bats (*Hypsignathus monstrosus, Epomops franqueti,* and *Myonycteris torquata)* captured in Gabon and the Democratic Republic of Congo (*5*). In one study, fruit and insectivorous bats (*Tadarida condylura, Tadarida pumila,* and *Epomophorus wahlbergi*) experimentally infected with EBOV were found to support the replication of the virus without apparent illness (*6*). In addition, researchers were able to culture EBOV from the sera and viscera of experimentally infected fruit bats (*6*). Exposure to fruit bats was implicated in an outbreak of EBOV in Luebo, Democratic Republic of Congo, in 2007 (*7*). However, EBOV has not been cultured from naturally infected bats.

Nonhuman primates (e.g., chimpanzees and gorillas) and duikers are susceptible to EBOV infection resulting in disease and death. EBOV has been detected by either nucleic acid amplification, immunohistochemical staining, or antigen detection from the carcasses of chimpanzees, gorillas, and duikers. In addition, contact with these animals has been directly implicated in several human outbreaks of EBOV (*7*,*8*). For other outbreaks, the animal source was unknown (*7*).

### Ebola Virus Disease in Humans

The incubation period for EVD is 2–21 days (mean: 4–10 days) (*1*). During the incubation period, the infected person is asymptomatic and cannot transmit the virus. After the incubation period, the infected person experiences illness, which usually begins with “dry symptoms” (e.g., fever, headache, muscle aches, or joint pain). This is followed by the onset of “wet symptoms” (e.g., nausea, vomiting, and diarrhea) at approximately day 4 of illness (*1*,*9*). The diarrhea can be severe, with outputs reported to be as high as 10 L/day, leading to severe metabolic derangements (*10*). Hemorrhage occurs in approximately half of patients and, when present, usually appears late in the disease course. Hemorrhagic manifestations can include bleeding from injection sites, epistaxis, hematemesis, hematochezia, melena, and gingival bleeding. Other signs and symptoms of EVD can include cough, shortness of breath, conjunctival injection, and an erythematous maculopapular rash. Laboratory findings in EVD include leukopenia, lymphopenia, and elevated transaminase levels. Depending on the severity of fluid losses, severe metabolic derangements also can be found. In persons who develop disseminated intravascular coagulation, elevated prothrombin and partial thromboplastin times and detectable fibrin-split products have been reported. Persons with fatal disease typically die 7–10 days after illness onset due to multisystem organ failure (*11*–*14*).

In persons who are infected, EBOV can be detected in all body fluids including urine, blood, saliva, sweat, feces, vomitus, semen, vaginal secretions, and breast milk. Person-to-person transmission of the virus occurs through direct contact with the body fluids of a person who is infected or has died of EVD. The virus can also be transmitted indirectly through contact with surfaces and objects contaminated with the body fluids of a person who is infected or has died of EVD (*15*,*16*).

### Clinical Sequelae of Ebola Virus Disease

Sequelae have been reported after infection with EBOV (*17*–*20*). After the 2014–2016 Ebola outbreak in West Africa, a long-term longitudinal study followed EVD survivors (n = 860) and EVD-negative close contact controls (n = 2,053) for 12 months. In this cohort, six symptoms were reported significantly more often among survivors than among controls: urinary frequency, headache, fatigue, muscle pain, memory loss, and joint pain (*20*). Ocular complaints, including photophobia, hyperlacrimation, conjunctivitis, and uveitis with subsequent cataract formation, also have been reported in survivors (*21*–*23*). In addition, a retrospective nationwide cohort study among survivors of the 2014–2016 West Africa Ebola outbreak in Guinea, conducted from December 8, 2015, through September 30, 2016, found that EVD survivors had a more than five times increased risk for death 1 year after recovery from the acute illness compared with the general Guinean population (age-standardized mortality ratio: 5.2; 95% confidence interval [CI]: 4.0–6.8) (*24*). Follow-up investigations using medical records and symptom checklists with family members suggested that renal failure might have contributed to approximately 60% of these deaths (*24*).

In survivors, EBOV has been known to enter and persist in immune-privileged sites such as the eye, placenta, testes, and central nervous system (*23*,*25*–*37*). EBOV has been cultured from ocular aqueous humor at 3 months after illness onset (*23*). EBOV can cross the placenta and has been detected by rRT-PCR in amniotic fluid, fetal meconium, umbilical cord, and buccal swab samples from neonates born to infected pregnant women (*33*). In addition, EBOV can persist in amniotic fluid for an unknown duration after clearance of the virus from maternal blood (*26*). EBOV RNA has been detected by rRT-PCR in the vaginal secretions of convalescent survivors (*38*,*39*). However, no transmission of the virus through female-to-male sexual contact has been documented (*40*).

EBOV has been shown to persist in semen after clinical recovery (*25*,*27*–*31*,*34*–*37*,*41*). EBOV has been cultured from semen up to 82 days after illness onset (*27*). Several studies demonstrated that EBOV was detected by rRT-PCR in semen of men within 3 months of their discharge from an Ebola treatment unit (ETU), with most men clearing the virus from the semen within 1 year (*34*–*36*). In a subset of men, the virus was shown to persist in semen >1 year from ETU discharge (*28*,*35*,*37*,*41*), with one study documenting rRT-PCR detection of EBOV as long as 965 days after illness onset (*28*). The factors associated with long-term persistence of EBOV RNA in semen are unknown. Age was suggested as a possible host determinant for EBOV RNA persistence in semen, with one study finding that EBOV was more likely to be detected by rRT-PCR in semen from older men (aged >40 years) after the first 3 months of recovery (chi-squared test, p = 0.0004) (*37*). The presence of immunocompromising disorders also has been suggested to be a factor, with EBOV RNA detected for up to 565 days after ETU discharge in an EVD survivor living with human immunodeficiency virus (HIV) (*41*).

Sexual transmission of EBOV was documented during the 2014–2016 Ebola outbreak in West Africa. The first documented occurrence was reported in November 2015 in Liberia (*29*). In this instance, a male EVD survivor transmitted the virus to a female sexual partner 199 days after illness onset. Although no infectious virus was isolated from the semen, genetic sequences of EBOV nucleic acid detected from the woman and from the semen of the male survivor closely matched (*29*,*30*). In February 2016, a male EVD survivor in Guinea sexually transmitted EBOV to his female partner 470 days after onset of symptoms. This transmission event resulted in a cluster of 13 infected persons in Guinea and Liberia (*31*).

One instance of disease recrudescence has been well documented in the published literature. Nine months after recovery from the acute illness, an EVD survivor had meningoencephalitis (*32*). EBOV was recovered from the cerebrospinal fluid and detected by rRT-PCR in blood 10 months after the initial EBOV infection (*32*).

Finally, EBOV has been cultured from breast milk up to 15 days after illness onset (*15*) and detected by rRT-PCR as long as 16 months after illness onset (*25*). In one published report from the 2014–2016 Ebola outbreak in West Africa, genomic analysis strongly suggested maternal-child transmission of the virus through infected breast milk (*42*).

### Treatment

Early supportive care (e.g., intravenous fluids, electrolyte supplementation, and nutritional support) alone can reduce mortality rates to approximately 40% (*14*,*43*–*45*). In 2018, a randomized control trial was launched in the Democratic Republic of Congo that evaluated three experimental therapies: remdesivir, an antiviral agent; mAb114, a single monoclonal antibody; and REGN-EB3, a triple monoclonal antibody, with ZMapp, a triple monoclonal antibody used during the 2014–2016 Ebola outbreak in West Africa, serving as the control. The primary endpoint of the study was death at 28 days. The study found that both mAb114 and REGN-EB3 were superior in reducing mortality rates due to EVD, by 35% and 33%, respectively. Additional reductions in deaths were observed in patients with low viral loads with mortality rates of 9.9% and 11.2%, respectively (*46*). The study also demonstrated the importance of early treatment, with an 11% increase in the odds of death for each day that symptoms persisted before study enrollment. On October 14, 2020, FDA approved Inmazeb (REGN-EB3) for the treatment of EVD caused by species *Zaire ebolavirus* in adult and pediatric patients (https://www.accessdata.fda.gov/drugsatfda_docs/label/2020/761169s000lbl.pdf).

### Ebola Virus Disease in the United States

A total of 11 persons infected with EBOV have been treated in the United States; all cases were associated with the 2014–2016 Ebola outbreak in West Africa (*13*,*43*,*47*–*49*). Nine cases were imported; one imported case resulted in secondary transmission of EBOV to two persons in the United States (*13*,*47*–*50*). Of the 11 persons infected with EBOV and treated in the United States, two died. In addition, during both the 2014–2016 Ebola outbreak in West Africa and the 2018 outbreak in North Kivu, South Kivu, and Ituri provinces in the Democratic Republic of Congo, additional persons were repatriated to the United States after high-risk exposures to confirmed EVD patients; none tested positive for EBOV (CDC, unpublished data, 2020).

### Groups at Highest Potential Occupational Risk for Ebola Virus Disease in the United States

The following are the three U.S. populations at highest risk for potential occupational exposure to EBOV:

- **Persons responding to an EVD outbreak.** Of the 11 persons infected with EBOV and treated in the United States, eight were responding to an outbreak.
- **Health care personnel at federally designated Ebola treatment centers in the United States.** As of December 2020, the United States has 11 federally designated Ebola treatment centers (Box). These specialized facilities are capable of caring for patients with high-consequence communicable diseases, such as viral hemorrhagic fevers. Four of these facilities treated patients with confirmed EVD during the 2014–2016 Ebola outbreak in West Africa. In total, an estimated 500 health care personnel work at the 11 federally designated Ebola treatment centers in the United States.
- **Laboratorians or other staff at biosafety level 4 facilities in the United States.** As of December 2020, the United States has 10 biosafety level 4 facilities (Box). Of these, eight facilities handle replication-competent EBOV. An estimated 400 laboratorians and support staff work at these eight facilities.

As of December 2020, Ervebo is the only vaccine licensed by the FDA for prevention of EVD caused by EBOV. Before Ervebo licensure, ACIP had no recommendations for the use of vaccines to prevent EVD. This report provides recommendations for use of Ervebo in the three U.S. populations at highest risk for potential occupational exposure to EBOV. Recommendations for use in additional populations and other settings will be considered and discussed by ACIP in the future.

## Methods

Beginning in September 2019, the ACIP Ebola Vaccine Work Group participated in bimonthly, and eventually weekly, conference calls. According to the Grading of Recommendations Assessment, Development and Evaluation (GRADE) approach (https://www.cdc.gov/vaccines/acip/recs/grade), the work group defined the research question (i.e., the patient, intervention, comparator, and outcome question), identified critical patient-centered outcomes, conducted a systematic review of the evidence, assessed the certainty of the evidence, and developed policy options for ACIP’s consideration. The work group identified the following critical outcomes of interest: development of symptomatic EVD, incidence and severity of vaccine-related joint pain or swelling, vaccine-related adverse pregnancy outcomes (defined as spontaneous abortion or stillbirth) for women inadvertently vaccinated while pregnant and women who became pregnant within 2 months of vaccination, serious adverse events related to vaccination, and transmissibility of vaccine virus from vaccinated individuals to nonvaccinated persons or animals. Summaries of evidence, including the GRADE evidence profiles (https://www.cdc.gov/vaccines/acip/recs/grade/ebola-vaccine.html) and evidence to recommendations (EtR) framework (https://www.cdc.gov/vaccines/acip/recs/grade/ebola-vaccine-etr.html), were presented at the February 2020 ACIP meeting. After a public comment period, ACIP voting members unanimously approved vaccination recommendations for three adult (aged ≥18 years) populations in the United States that are at the highest risk for potential occupational exposure to EBOV and for whom preexposure vaccine recommendations are the most urgent: persons responding to an outbreak of EVD, health care personnel at federally designated Ebola treatment centers in the United States (Box), and laboratorians or other staff at biosafety level 4 facilities in the United States (Box).

## Summary of Findings

### Vaccine Efficacy

Vaccine efficacy was evaluated in a two-part phase 3, open-label, cluster-randomized, controlled ring vaccination trial in Guinea during the 2014–2016 Ebola outbreak in West Africa (*51*,*52*). In the initial study, clusters of contacts of confirmed EVD patients and contacts of those contacts were offered immediate vaccination or delayed vaccination (21 days after randomization). The primary outcome of interest was incidence of laboratory-confirmed EVD with onset ≥10 days after randomization. The 10-day period was selected to account for the incubation period of EVD and the unknown length of time from vaccination to the development of protective immunity. Analysis of the randomized clusters in the initial study demonstrated that among the 2,108 participants vaccinated immediately, none developed EVD ≥10 days after randomization. In the delayed group, 10 of 1,429 participants developed EVD ≥10 days after randomization. On the basis of the cluster-level data, vaccine efficacy in the initial study was calculated to be 100% (95% CI: 63.5%–100%) in participants receiving immediate vaccination.

The follow-up study included additional data about clusters of contacts and contacts of contacts who were offered immediate vaccination. Analysis of the randomized and nonrandomized clusters in the follow-up study demonstrated that no one who was immediately vaccinated (0 of 3,775 participants) developed EVD ≥10 days after randomization (or for nonrandomized participants, the date of inclusion in the ring). In the delayed or never-vaccinated group, 23 of 4,507 participants developed EVD ≥10 days after randomization. On the basis of cluster-level data, vaccine efficacy in the follow-up study was calculated to be 100% (95% CI: 79.3%–100%).

### Vaccine Safety

Three types of joint-related adverse events after vaccination were reviewed: vaccine-related arthralgia with onset 0–42 days postvaccination, severe arthralgia with onset 0–42 days postvaccination, and vaccine-related arthritis with onset 5–56 days postvaccination. Joint-related adverse events were more commonly reported among vaccine recipients compared with nonvaccinated persons. Additional details regarding the methods used to assess vaccine safety are available (https://www.cdc.gov/vaccines/acip/recs/grade/table-refs.html).

#### Arthralgia with Onset 0–42 Days Postvaccination

Typically, arthralgia was reported within a few days of vaccination, was mild to moderate in intensity, and resolved within 1 week after onset (*53*–*58*). The reported incidence of arthralgia, regardless of severity, in the studies evaluated by GRADE ranged from 2.9% to 40%; however, incidence varied substantially among studies depending on the definition of arthralgia and the method and timing of ascertainment (*53*,*57*). A meta-analysis of six randomized trials included in the body of evidence for this outcome found an overall relative risk (RR) of 2.55 (95% CI: 0.94–6.91) and an absolute risk (AR) of 73 more cases of arthralgia per 1,000 persons vaccinated (95% CI: 3 fewer to 279 more) (*53**–**58*).

Vaccine virus was detected by rRT-PCR in the synovial fluid of four of seven vaccinated participants with joint complaints whose synovial fluid was tested (*56*,*58*,*59*). Virus isolation was attempted on one of these synovial fluid specimens and was negative (*59*).

#### Severe Arthralgia with Onset 0–42 Days Postvaccination

Severe (grade 3) arthralgia was defined as significant joint pain or discomfort that prevents daily activity. The reported incidence of severe arthralgia in the studies evaluated by GRADE ranged from 0% to 1.7%; however, incidence varied substantially among studies depending on the method and timing of ascertainment (*53*–*55*,*58*,*60*). A meta-analysis of four randomized trials included in the body of evidence for this outcome found the overall RR was 6.40 (95% CI: 0.0001–27,950.69) and an AR of zero fewer cases of severe arthralgia per 1,000 persons vaccinated (95% CI: 0 to 0 fewer) (*53**–**55**,**58*).

#### Arthritis with Onset 5–56 Days Postvaccination

Typically, arthritis was reported 1 week after vaccination and resolved within days to weeks after onset (*58*–*60*). The reported incidence of arthritis in the studies evaluated by GRADE ranged from 0% to 23.5%; however, incidence varied substantially among studies depending on the definition of arthritis and the method and timing of ascertainment (*53*,*58*). Some vaccinated persons experienced recurrent or prolonged joint symptoms (*58*,*59*). All joint types (i.e., small and large joints as well as the axial skeleton) were reported to be affected (*58*,*59*). A meta-analysis of four randomized trials included in the body of evidence for this outcome found the overall RR was 1.80 (95% CI: 0.21–15.13); AR was 23 more cases of arthritis per 1,000 persons vaccinated (95% CI: 22 fewer to 400 more) (*53*,*55*–*57*). One post hoc multivariate logistic regression analysis identified female sex and a medical history of arthritis to be independent factors associated with a 2.2-fold to 2.8-fold higher risk for developing postvaccination arthritis (95% CIs: 1.1–4.1 and 1.3–6.2, respectively) (*61*).

#### Vaccine-Related Adverse Pregnancy Outcomes

Vaccine-related adverse pregnancy outcomes were assessed with respect to the incidence of pregnancy loss, defined as spontaneous abortion or stillbirth, in women inadvertently vaccinated while pregnant and women who became pregnant within 2 months of vaccination. The body of evidence for this outcome is from one study (*62*). In this unblinded phase 2/3 clinical trial, participants were randomly assigned to immediate or deferred vaccination groups. Pregnancy was an exclusion criterion and, before enrollment, women of reproductive age (18–49 years) were asked about pregnancy status and were required to take a urine pregnancy test. After vaccination, women were advised to avoid pregnancy for 60 days from the time of vaccination. A total of 84 women were inadvertently vaccinated in early pregnancy or became pregnant ≤60 days from vaccination or enrollment. Among immediately vaccinated pregnant women, 14 of 31 (45%) experienced pregnancy loss compared with 11 of 33 (33%) unvaccinated pregnant women (unadjusted RR: 1.35; 95% CI: 0.73–2.52). Overall, the rate of pregnancy loss among pregnant women who received immediate vaccination was not statistically significantly higher than the rate of pregnancy loss among unvaccinated pregnant women. No external congenital anomalies were detected among live-born infants in either group (n = 44).

#### Transmissibility of Vaccine Virus to Susceptible Humans or Animals

No data are available on the transmissibility of vaccine virus (rVSVΔG-ZEBOV-GP) to susceptible humans and animals after vaccination with Ervebo. Therefore, detection of vaccine virus RNA in a vaccine recipient’s blood, saliva, and urine by rRT-PCR was used as a surrogate for potential vaccine virus transmissibility. Eight studies reported the detection of vaccine virus RNA in blood by rRT-PCR (*53*,*54*,*57*–*60*,*63*,*64*). The longest recorded rRT-PCR–positive sample was collected 14 days postvaccination (*60*). Four studies reported the detection of vaccine virus RNA in saliva and urine by rRT-PCR (*53*,*54*,*59*,*60*). The longest recorded rRT-PCR–positive saliva sample was collected 14 days postvaccination (*54*). The longest recorded rRT-PCR–positive urine sample was collected 7 days postvaccination (Table) (*54*,*60*). Virus isolation was performed in a subset of rRT-PCR–positive blood samples from one study; all were negative (*59*). No data are available on transmissibility of the vaccine virus through sexual contact.

**TABLE:** Detection of recombinant vesicular stomatitis virus–vaccine virus in persons vaccinated with Ervebo

Specimen type	Vaccine virus detected by rRT-PCR	Longest recorded positive rRT-PCR (dpv)	Culture of vaccine virus attempted	Vaccine virus detected by culture
Blood	Yes	14*	Yes	No
Urine	Yes	7*	No	Not performed
Saliva	Yes	14*	No	Not performed
Synovial fluid	Yes	17^†^	Yes^§^	No^§^
Skin vesicles	Yes	20^¶^	Yes	Yes**

**Abbreviation:** dpv = days postvaccination; rRT-PCR = reverse transcriptase–polymerase chain reaction.

* One study also tested at 28 dpv and none of 38 were positive (**Source:** Heppner DG Jr, Kemp TL, Martin BK, et al; V920-004 Study Team. Safety and immunogenicity of the rVSV∆G-ZEBOV-GP Ebola virus vaccine candidate in healthy adults: a phase 1b randomised, multicentre, double-blind, placebo-controlled, dose-response study. Lancet Infect Dis 2017;17:854–66).

^†^ Of three participants in one study, whose synovial fluid was tested by rRT-PCR, one tested positive 17 dpv and two tested negative 16 and 23 dpv (**Source:** Halperin SA, Arribas JR, Rupp R, et al; V920-012 Study Team. Six-month safety data of recombinant vesicular stomatitis virus–Zaire Ebola virus envelope glycoprotein vaccine in a phase 3 double-blind, placebo-controlled randomized study in healthy Adults. J Infect Dis 2017;215:1789–98).

^§^ One synovial fluid specimen tested positive by rRT-PCR 15 dpv and virus isolation was negative (**Source:** Agnandji ST, Huttner A, Zinser ME, et al. Phase 1 trials of rVSV Ebola vaccine in Africa and Europe. N Engl J Med 2016;374:1647–60).

^¶^ **Source:** Ervebo [Package Insert]. Whitehouse Station, NJ: Merck; 2009. https://www.fda.gov/media/133748/download

** Of three participants with rash in one study, all were positive by rRT-PCR up to 17 dpv, and virus isolation was positive in one specimen with the highest RNA level 9 dpv (**Source:** Agnandji ST, Huttner A, Zinser ME, et al. Phase 1 trials of rVSV Ebola vaccine in Africa and Europe. N Engl J Med 2016;374:1647–60).

#### Vaccine-Related Serious Adverse Events

Overall, reported vaccine-related serious adverse events were rare. Across 12 clinical trials, out of 15,399 persons who received the vaccine, three serious adverse events were judged to be related or possibly related to the vaccine: one febrile reaction, one anaphylactic reaction, and one influenza-like illness (*51*,*53*–*55*,*58*–*61*,*63*,*64*). All resolved without sequelae (*51*). An additional case of anaphylaxis was identified in data provided to FDA and resolved without sequelae (*51*,*65*,*66*). Two additional publications describing expanded access use of the vaccine reported on the vaccination of 1,720 individuals with zero serious adverse events (*67*,*68*).

## Rationale for Recommendations

EVD is a highly transmissible disease with high morbidity and mortality. Ervebo is the only vaccine licensed by FDA for the prevention of EVD due to EBOV. In outbreak settings, the vaccine has been shown to be efficacious. Licensure of the vaccine offers an opportunity to prevent EVD in U.S. populations at highest risk for potential occupational exposure. Joint-related adverse events have been reported in some vaccinated persons. The reported incidence of these joint-related adverse events has varied substantially among studies because of differences in case definition and the method and timing of ascertainment; however, the range has been 2.9%–40% for arthralgia (*53*,*57*), 0%–1.7% for severe arthralgia (*53*–*55*,*58*,*60*), and 0%–23.5% for arthritis (*53*,*58*) for studies included in the GRADE assessment. With the detection of vaccine virus in synovial fluid (*56*,*58*,*59*) in a proportion of vaccinated persons with joint complaints, these joint-related adverse events are likely to be vaccine related (Table). The decision to recommend preexposure vaccination in these groups is based on the following reasons: documented protective efficacy of the vaccine against the development of symptomatic EVD, high mortality and severity of illness in persons infected with EBOV, high transmissibility of EBOV, EVD-related sequelae in survivors and the potential for continued disease transmission and disease recrudescence, lack of an FDA-approved treatment for EVD at the time the recommendations were made, and an acceptable safety profile relative to the severity of EBOV infection.

## Recommendations

Preexposure vaccination with Ervebo is recommended for adults aged ≥18 years in the U.S. population who are at highest risk for potential occupational exposure to EBOV because they are

- responding to an outbreak of EVD,
- working as health care personnel at federally designated Ebola treatment centers in the United States (Box), or
- working as laboratorians or other staff at biosafety level 4 facilities in the United States (Box).

## Clinical Guidance

### Vaccine Storage and Handling

 The vaccine is supplied as single-dose vials containing a 1-mL dose and must be stored frozen at −112°F to −76°F (−80°C to −60°C) for long-term storage. To administer the vaccine, thaw the vial at room temperature until no visible ice is present. The vaccine should be administered immediately after thawing. However, if the vaccine is not used immediately, the thawed vial may be stored at 35.6°F–46.4°F (2°C–8°C) for a maximum of 2 weeks and up to 4 hours at room temperature (up to 77°F [25°C]), protected from light. Additional information is available in the package insert (https://www.fda.gov/media/133748/download).

### Dosage and Administration

Ervebo should be administered as a single 1-mL intramuscular dose, preferably in the deltoid area of the nondominant arm. The vaccine should not be mixed in the same syringe with any other vaccines or medicinal products. The vaccination injection site should be covered with a bandage that provides a physical barrier to protect the site from direct contact. The bandage can be removed when there is no visible fluid leakage. Contaminated bandages should be placed in a sealed plastic bag and disposed of in the trash. Hands should be washed with soap and water after disposing the plastic bag. Additional information is available in the package insert (https://www.fda.gov/media/133748/download).

### Adverse Reactions

The most commonly reported injection-site adverse events were pain, swelling, and redness (*66*). The most commonly reported systemic adverse events were headache (37%), fever (34%), muscle pain (33%), fatigue (19%), joint pain (18%), nausea (8%), arthritis (5%), rash (4%), and abnormal sweating (3%) (*66*).

Some vaccinated persons might experience arthralgia and arthritis after vaccination. Typically, arthralgia began within 1–2 days after vaccination, was mild to moderate in intensity, and resolved within 1 week after onset (*53**–**58*). Typically, arthritis began 1 week after vaccination and resolved within days to weeks after onset (*58*–*60*). However, some vaccinated persons reported prolonged and recurrent symptoms (*58*,*59*). One post hoc analysis identified women and persons with a medical history of arthritis to have a 2.2-fold to 2.8-fold higher risk for developing postvaccination arthritis (*61*).

### Management of Acute Allergic Reactions

Anaphylaxis has been observed after administration of the vaccine. Monitor persons for signs and symptoms of hypersensitivity reactions for 15 minutes after vaccination. Appropriate medical treatment must be available in case of an anaphylactic event (https://www.cdc.gov/vaccines/hcp/acip-recs/general-recs/adverse-reactions.html).

### Special Populations

#### Pregnant Women

Human data available from clinical trials with Ervebo are insufficient to establish the presence or absence of vaccine-associated risk during pregnancy. The decision regarding whether to vaccinate a pregnant woman should involve consideration of the woman’s risk for exposure to EBOV (*62*).

#### Lactating Women

Human data are not available to assess the impact of Ervebo on milk production, its presence in breast milk, or its effects on the breastfed child. The development and health benefits of breastfeeding should be considered along with the mother’s clinical need for vaccination and any potential adverse effects on the breastfed child from Ervebo. The decision regarding whether to vaccinate a lactating woman should involve consideration of the woman’s risk for exposure to EBOV.

#### Immunocompromised Persons

Safety and efficacy of Ervebo have not been adequately assessed in immunocompromised adults. A small number of adults living with HIV have been vaccinated with Ervebo (*57*). Additional studies are ongoing to investigate use of Ervebo in persons living with HIV without severe immune compromise (https://clinicaltrials.gov/ct2/show/NCT03031912?term=ebola+vaccine&draw=3&rank=19). The risk from vaccination with Ervebo, a live virus vaccine, in immunocompromised persons should be weighed against the risk for disease due to EBOV.

### Contraindications: Hypersensitivity or Allergy to Vaccine Components

Ervebo is contraindicated for persons with a history of severe allergic reaction (e.g., anaphylaxis) to any component of the vaccine. Because Ervebo contains rice-derived recombinant human serum albumin, caution should be taken with persons allergic to rice. Persons with a history of severe allergic reaction (e.g., anaphylaxis) to rice protein should not receive Ervebo.

### Precautions: Vaccine Virus Transmission

Ervebo contains a replication-competent, live attenuated virus; therefore, potential exists for transmission of vaccine virus through close personal contact. Vaccine virus RNA has been detected by rRT-PCR in the blood (*60*) and saliva (*54*) of vaccinated persons as long as 14 days after vaccination. Vaccine virus RNA has been detected in the urine of vaccinated persons as long as 7 days after vaccination (*54*,*60*). Vaccine virus RNA has been detected by rRT-PCR in synovial fluid and skin vesicles in vaccinated persons as long as 20 days after vaccination (*66*). Live vaccine virus has been isolated in skin vesicles in vaccinated persons as long as 9 days after vaccination (*59*) (Table).

The following precautions should be taken to prevent potential vaccine virus transmission:

- Vaccine recipients should not donate blood for at least 6 weeks postvaccination (*69*).
- Vaccine recipients should avoid sharing needles, razors, or eating utensils; drinking from the same cup; using the same toothbrushes; and open-mouth kissing for 2 weeks after vaccination. Persons who develop oral sores after receiving vaccine should avoid these activities until the sores heal.
- Out of an abundance of caution, vaccine recipients should use effective barrier prophylaxis methods during any sexual interaction (regardless of childbearing status or sexual orientation) for 2 months after vaccination (*69*).
- Vaccine recipients should consider avoiding close association with and exposure of high-risk persons to their blood and bodily fluids for up to 6 weeks after vaccination. High-risk persons include immunocompromised persons, pregnant or breastfeeding women, and children aged <1 year (*69*).
- Vaccine recipients should attempt to avoid exposing livestock to their blood and bodily fluids for at least 6 weeks after vaccination (*69*).
- Both maculopapular and vesicular rashes have been described in some individuals after vaccination. Persons who develop a rash after receiving the vaccine should cover the rash with a bandage until healed. Contaminated bandages should be placed in a sealed plastic bag and disposed of in the trash. Hands should be washed with soap and water after disposing the plastic bag.

### Limitations of Vaccine Effectiveness

The duration of protection against EBOV after vaccination with Ervebo is unknown. In addition, vaccination with Ervebo might not protect all persons. Vaccinated persons should continue to adhere to recommended infection control practices to prevent EBOV infection and transmission among health care workers (https://www.cdc.gov/vhf/ebola/clinicians/evd/infection-control.html) and laboratorians (https://www.cdc.gov/labs/BMBL.html).

### Impact on Ebola Serological Testing

After vaccination with Ervebo, persons might test positive for the EBOV glycoprotein. The EBOV glycoprotein is a target for certain serologic and molecular Ebola diagnostic tests. Therefore, diagnostic testing for EVD in vaccinated persons also should include nonglycoprotein targets (*66*).

###  Vaccine Availability

Ervebo is not commercially available in the United States. The vaccine is stored and made available for U.S. civilians at no cost through the U.S. government in accordance with ACIP recommendations on receipt of request. The vaccine should be administered under the supervision of a physician. Vaccine is shipped to the responsible physician. For information on how to submit vaccine requests, refer to https://www.cdc.gov/laboratory/drugservice/formulary.html or contact CDC’s Viral Special Pathogens Branch at spathvax@cdc.gov.

### Reporting of Adverse Events after Vaccination

As with any newly licensed vaccine, surveillance for adverse events associated with administration of Ervebo is important for assessing its safety. All clinically significant adverse events should be reported to Vaccine Adverse Events Reporting System (VAERS) at https://vaers.hhs.gov, even if causal relation to vaccination is unknown or not certain. Reports to VAERS can be made electronically, either online or by fillable PDF form (https://vaers.hhs.gov/esub/index.jsp), or by telephone (800-822-7967). Providers are encouraged to report electronically to promote better timeliness and quality of safety data.

## Future Research

Research is ongoing in several areas, including safety of Ervebo in immunocompromised persons, pregnant women, and children. In addition, long-term studies are underway to assess immunogenicity and duration of protection. ACIP will consider these data as they become available and revise recommendations accordingly. In addition, the Ebola Vaccine Work Group will continue to identify and discuss recommendations for additional populations at potential occupational risk.

As with all new vaccines, CDC will monitor adverse events after Ervebo administration through VAERS and the Vaccine Safety Datalink. Managed by CDC and FDA, VAERS is a national early warning system to monitor the safety of U.S.-licensed vaccines. Additional information about VAERS is available (https://vaers.hhs.gov/about.html). The Vaccine Safety Datalink monitors the safety of vaccines and possible adverse events when new vaccines are licensed or when new vaccine recommendations are published. Additional information about the Vaccine Safety Datalink is available (https://www.cdc.gov/vaccinesafety/ensuringsafety/monitoring/vsd/index.html). Additional postmarketing safety monitoring will include studies conducted by Merck and reported to FDA.

## Footnotes

* Health care personnel refers to all paid and unpaid persons serving in health care settings who have the potential for direct or indirect exposure to patients or infectious materials, including body substances (e.g., blood, tissue, and specific body fluids); contaminated medical supplies, devices, and equipment; contaminated environmental surfaces; or contaminated air. These health care personnel include emergency medical service personnel, nurses, nursing assistants, physicians, technicians, clinical laboratory personnel, autopsy personnel*,* therapists, phlebotomists, pharmacists, students and trainees, contractual staff not employed by the health care facility, and persons not directly involved in patient care but who could be exposed to infectious agents that can be transmitted in the health care setting (e.g., clerical, dietary, environmental services, laundry, security, engineering and facilities management, administrative, billing, and volunteer personnel) (**Source:** https://www.cdc.gov/infectioncontrol/guidelines/healthcare-personnel/index.html).

† The denominator of 32 does not include laboratory accidents or imported cases.
